# Quantitative and Qualitative Analysis of Bone Marrow CD8^+^ T Cells from Different Bones Uncovers a Major Contribution of the Bone Marrow in the Vertebrae

**DOI:** 10.3389/fimmu.2015.00660

**Published:** 2016-01-13

**Authors:** Sulima Geerman, Sarah Hickson, Giso Brasser, Maria Fernanda Pascutti, Martijn A. Nolte

**Affiliations:** ^1^Department of Hematopoiesis, Sanquin Research, Amsterdam, Netherlands; ^2^Landsteiner Laboratory, Academic Medical Center, University of Amsterdam, Amsterdam, Netherlands

**Keywords:** vertebrae, bone marrow, CD8^+^ T cells, memory, LCMV, CD69, tissue-resident, Ki-67

## Abstract

Bone marrow (BM) plays an important role in the long-term maintenance of memory T cells. Yet, BM is found in numerous bones throughout the body, which are not equal in structure, as they differ in their ratio of cortical and trabecular bone. This implies that BM cells within different bones are subjected to different microenvironments, possibly leading to differences in their frequencies and function. To address this, we examined BM from murine tibia, femur, pelvis, sternum, radius, humerus, calvarium, and the vertebrae and analyzed the presence of effector memory (T_EM_), central memory (T_CM_), and naïve (T_NV_) CD8^+^ T cells. During steady-state conditions, the frequency of the total CD8^+^ T cell population was comparable between all bones. Interestingly, most CD8^+^ T cells were located in the vertebrae, as it contained the highest amount of BM cells. Furthermore, the frequencies of T_EM_, T_CM_, and T_NV_ cells were similar between all bones, with a majority of T_NV_ cells. Additionally, CD8^+^ T cells collected from different bones similarly expressed the key survival receptors IL-7Rα and IL-15Rβ. We also examined BM for memory CD8^+^ T cells with a tissue-resident memory phenotype and observed that approximately half of all T_EM_ cells expressed the retention marker CD69. Remarkably, in the memory phase of acute infection with the lymphocytic choriomeningitis virus (LCMV), we found a massive compositional change in the BM CD8^+^ T cell population, as the T_EM_ cells became the dominant subset at the cost of T_NV_ cells. Analysis of Ki-67 expression established that these T_EM_ cells were in a quiescent state. Finally, we detected higher frequencies of LCMV-specific CD8^+^ T cells in BM compared to spleen and found that BM in its entirety contained fivefold more LCMV-specific CD8^+^ T cells. In conclusion, although infection with LCMV caused a dramatic change in the BM CD8^+^ T cell population, this did not result in noticeable differences between BM collected from different bones. Our findings suggest that in respect to CD8^+^ T cells, BM harvested from a single bone is a fair reflection of the rest of the BM present in the murine body.

## Introduction

The bone marrow (BM) acts as the primary site for the formation of all mature blood cells through the process of hematopoiesis. The complex hematopoietic process that gives rise to these cells takes place in the red (hematopoietic) part of the BM. At birth, BM primarily consists of red marrow, but with age, the red marrow decreases and is replaced by yellow (adipocytic) marrow (1). In adults (>25 years of age), red marrow is predominantly located in the tips (epiphysis), whereas yellow marrow is mostly found in the shafts (diaphysis) of the long bones. The epiphysis primarily consists of trabecular (spongy) bone, whereas the diaphysis consists of cortical (compact) bone (2). These differences in the composition of bone have been shown to influence the function of the BM. Human hematopoietic stem cells (HSCs) isolated from trabecular marrow of long bones have superior regenerative and self-renewal capacity compared to HSCs from the cortical marrow harvested from the shaft area and have also been shown to be molecularly distinct (3). Farrell et al. (4) found similar numbers of human HSCs and myeloid progenitor cells (GM-CFCs) in trochanter marrow (region between the epiphysis and diaphysis of the femur) compared to marrow in the femoral epiphysis. However, they observed a decline in numbers of femoral marrow-derived GM-CFCs in aged individuals, while the numbers for GM-CFCs derived from trochanter marrow did not change. In mice, substantial heterogeneity has been found in bone remodeling activity, blood volume fraction, and hypoxia between epiphysis, diaphysis, and calvarium, which were also shown to affect HSC function (5). These data indicate that distinct compartments within the BM are different, leading to functional differences for the cells that reside in these specific niches.

Next to its important function as a primary lymphoid organ, the BM has also gained recognition for its role as a secondary lymphoid organ. Dendritic cells in the BM can take up and present blood-borne antigens and thereby activate local naïve T cells (6). Neutrophils can capture and transport virus from the dermis into the BM, leading to priming of virus-specific CD8^+^ T cells by local antigen presenting cells (7). Additionally, the BM is also actively involved in immunological memory. Effector T cells that survive antigen clearance develop into memory T cells and home to the BM. Here, they provide life-long protection against reinfection (8–10). Studies in mice lacking IL-7 and IL-15 or their receptors IL-7Rα (CD127) and IL-15Rβ (CD122) have shown that these two cytokines are vital for the maintenance of memory CD8^+^ T cells, as they affect both their generation and survival (11–13). These effects could be direct, but they could also be mediated indirectly through the induction of costimulatory molecules that control memory T cell survival (14, 15). It has recently been shown that BM memory CD8^+^ T cells acquire IL-7 by docking to IL-7-producing reticular stromal cells (16). Additionally, human memory CD8^+^ T cells have been shown to be in close contact with a variety of IL-15-producing BM cells. These BM resident cells displayed morphological characteristics of stromal cells, dendritic cells, and monocytes (17).

Thus, it is now clear that BM is important for long-term memory maintenance and is therefore more frequently included in studies of (adaptive) immune responses [reviewed in Ref. (18, 19)]. However, little is known about quantitative and qualitative differences between various bones regarding T cell maintenance. Most information on BM T cells has been obtained from single cell suspensions prepared from crushed or flushed tibia and/or femurs. Hence, the BM has been conceptually and also practically regarded as a single organ. However, this view may not be justified, as bones throughout the body are diverse in their composition of cortical and trabecular bone depending on their mechanical or organ protection function (2), already leading to functional differences at the level of HSCs. Here, we examined if anatomical differences exist in BM, by assessing the CD8^+^ T cell population in BM harvested from murine tibia, femur, pelvis, sternum, radius, humerus, calvarium, and vertebrae and compared this to the spleen. We found that both in steady state and after infection with acute lymphocytic choriomeningitis virus (LCMV), BM located in distinct bones have comparable frequencies of CD8^+^ T cell subsets. Furthermore, by calculating the total number of BM CD8^+^ T cells found in the entire body, we demonstrate that BM is superior to spleen in harboring memory CD8^+^ T cells, and that this is attributed to the major contribution of the memory CD8^+^ T cells present in the vertebrae.

## Materials and Methods

### Mice

Wild-type (WT) C57BL/6J mice were kept under specific pathogen-free conditions in the animal facility of the Academic Medical Center (Amsterdam, The Netherlands) or Netherlands Cancer institute (Amsterdam, The Netherlands). Female or male mice between the age of 13 and 17 weeks were used for steady-state experiments. For LCMV experiments, mice that were 9–16 weeks old were injected intraperitoneally with 2.0 × 10^5^ PFU of the Armstrong strain, kindly provided by Dr. Ramon Arens (LUMC, Leiden, The Netherlands) in 200 μl PBS. Mice were sacrificed during the memory phase (>42 days post injection). Mice received chow and acidified drinking water *ad libitum*. Animal experiments were performed in accordance with the institutional and national guidelines and approved by the Experimental Animal Committees of both animal facilities.

### Sample Collection and Preparation

Tibia, femur, pelvis, radius together with humerus were collected from every mouse. Additionally, we harvested sternum, calvarium, and the vertebrae (cervical vertebrae C1–sacral vertebrae S5). Bones were cleaned and crushed in MACS buffer (PBS + 1% FCS + 2 mM EDTA) using a mortar and pestle. BM cell suspensions were filtered through a 40-μm cell strainer to remove bone debris. Single splenocyte suspensions were prepared by crushing the spleen through a 40-μm cell strainer with the plunger of a syringe. For several LCMV experiments, whole spleen and BM cells were enriched for CD8^+^ T cells with CD8α microbeads (Miltenyi Biotec) and MACS LS columns (Miltenyi Biotec). Erythrocytes were lysed with red blood cell lysis buffer (155 mM NH_4_Cl, 10 mM KHCO_3_, 127 mM EDTA). White blood cells were counted with CASY Cell Counter and Analyzer (Roche).

### Flow Cytometry Analysis

The following antibodies were used in this study: CD3ε-eFlour 450 (145-2C11, eBioscience), CD3ε-APC-eFluor 780 (17A2, eBioscience), CD8α-APC-eFluor 780 (53-6.7, eBioscience), CD8α-BV605 (53-6.7, Biolegend), CD8α-PerCP-Cy5.5 (53-6.7, eBioscience), CD44-PE-Cy7 (IM7, Biolegend), CD62L-APC (MEL-14, eBioscience), CD62L-BV510 (MEL-14, Biolegend), CD69eFluor 450 (H1.2F3, eBioscience) CD69-biotin (H1.2F3, eBioscience), CD122-FITC (TM-B1, BD Biosciences), CD127-BV605 (A7R34, Biolegend), and Streptavidin PerCP-Cy5.5 (BD Biosciences). The MHC class I tetramers H2-D^b^GP_33–41_ APC and H2-D^b^NP_396–404_ PE were kind gifts from Dr. Ramon Arens (LUMC, Leiden, The Netherlands). Cells were fixed with Foxp3/Transcription Factor Staining buffer set (eBioscience) and stained with Ki-67 PE or Ki-67 FITC (B56, BD Biosciences). Samples were acquired with the LSR Fortessa (BD) and analyzed with FlowJo software (Tree Star, Inc.).

### Statistical Analysis

Statistical analyses were performed with Prism (GraphPad Software, Inc.) using an unpaired *t* test followed by Welch’s correction or a one-way ANOVA followed by Tukey’s correction. Significance is indicated by *p < 0.05, **p < 0.01, ***p < 0.001, ****p < 0.0001, and ^#^*p* < 0.05 between spleen and all the different bones.

## Results

### BM Contains More Memory CD8^+^ T Cells than the Spleen

**Figure 1:**
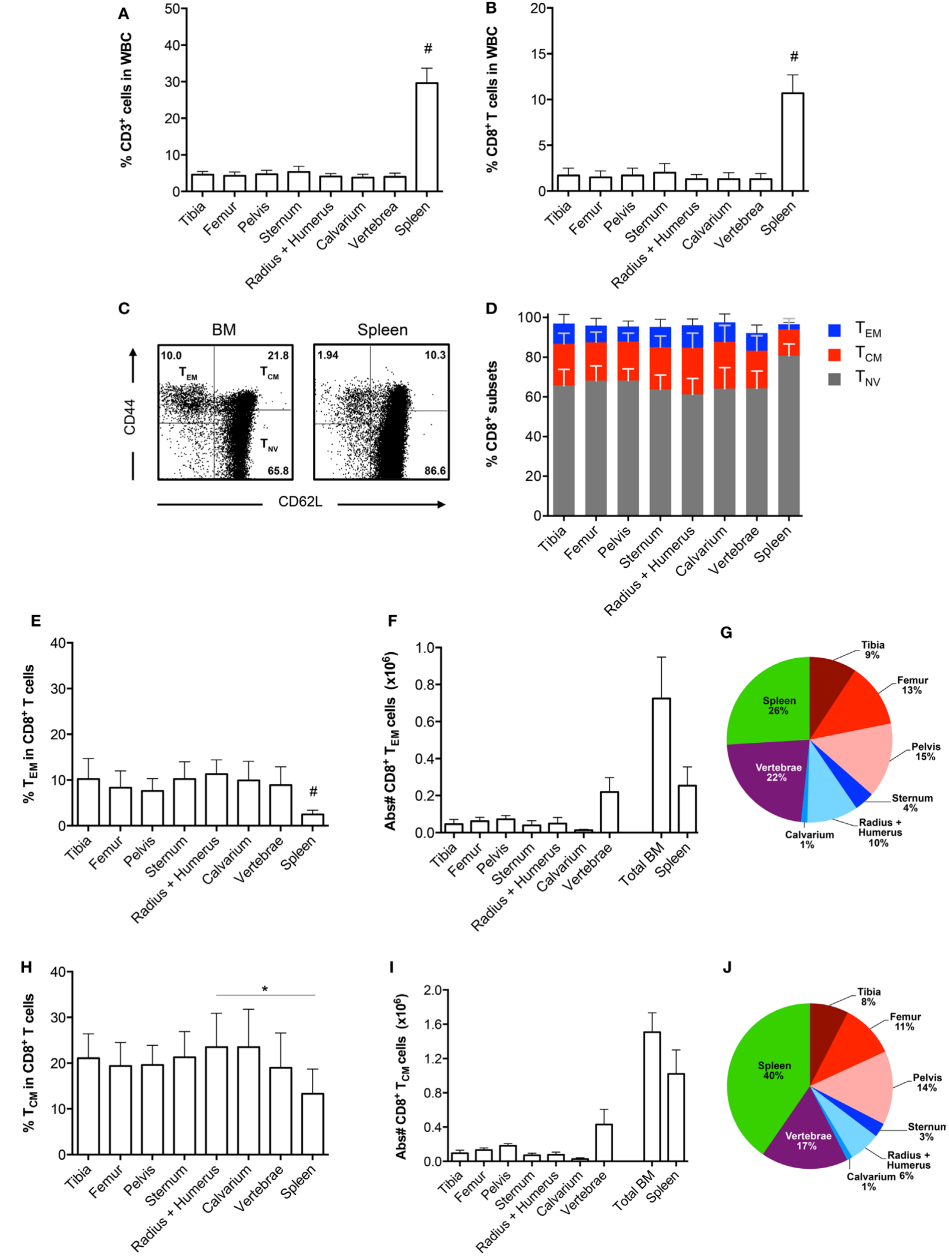
**Comparable CD8^+^ T cell frequencies in BM obtained from different bones**. **(A)** Frequency of CD3^+^ and **(B)** CD8^+^ T cells in total white blood cells (WBC). **(C)** Representative FACS plots showing expression of CD44 and CD62L in CD8^+^ T cells for BM (radius + humerus) and spleen. **(D)** Frequency of T_EM_, T_CM_, and T_NV_ cells within the total CD8^+^ T cell population. **(E)** Frequency and **(F)** absolute numbers of T_EM_ cells within the total CD8^+^ T cell population. **(G)** Distribution of T_EM_ cells (based on absolute numbers). **(H)** Frequency and **(I)** absolute numbers of T_CM_ cells within the total CD8^+^ T cell population. **(J)** Distribution of T_CM_ cells (based on absolute numbers). The double amount of cells was taken into account for estimating the contribution of tibia, femur, pelvis, and radius + humerus for the calculation of “Total BM” in **(F,I)** and for their contribution in **(G,J)**. Graphs show mean ± SD of each bone (*n* = 4–8), pooled from the three independent experiments. Statistical analysis was performed with a one-way ANOVA followed by Tukey’s correction. Significance is indicated by *p < 0.05 or ^#^*p* < 0.05 between spleen and all bones.

To examine whether the composition of the CD8^+^ T cell population in a single bone is representative of all other bones, we examined the CD8^+^ T cells in BM from tibia, femur, pelvis, sternum, radius, humerus, calvarium, and vertebrae and compared this to the spleen. We observed that the frequencies of CD3^+^ cells, and also of CD8^+^ T cells, were significantly lower in all bones compared to the spleen (Figures 1A,B). Regarding the presence of the classical CD8^+^ T cell subsets, i.e., effector memory (T_EM_; CD44^+^CD62L^−^), central memory (T_CM_; CD44^+^CD62L^+^), and naïve (T_NV_; CD44^−^CD62L^+^) T cells (20, 21), we found that all bones consisted primarily (~65%) of T_NV_ cells (Figures 1C,D). Strikingly, the frequencies of T_EM_, T_CM_, and T_NV_ cells between the different bones were highly comparable. We observed that in comparison to spleen, all bones had higher frequencies of CD8^+^ T_EM_ cells (Figure 1E). A similar pattern was observed for CD8^+^ T_CM_ cells (Figure 1H). In absolute numbers, the majority of BM memory CD8^+^ T cells was located in the vertebrae (Figures 1F,I), as these contained the highest amount of BM cells, almost equivalent to the spleen (Table 1). BM, in its totality, contained ~2.8-fold more T_EM_ and ~1.5 more T_CM_ cells compared to spleen (Figures 1G,J). Taken together, we conclude that in the steady state, BM is quite distinct from spleen regarding frequencies of CD3^+^ and CD8^+^ T cells, but comparable between the different bones. Additionally, we show that BM accumulates, also in absolute numbers, more memory CD8^+^ T cells compared to spleen.

**Table 1:** **BM yield from different bones**.

Absolute number of white blood cells (**×**10^6^)	
	Steady state (*n* = 5)
Tibia	13.7 ± 8.0
Femur	25.3 ± 7.5
Pelvis	25.6 ± 10.1
Sternum	7.9 ± 2.6
Radius + humerus	14.4 ± 2.2
Calvarium	6.0 ± 2.0
Vertebrae	97.5 ± 16.9
Spleen	99.3 ± 9.9

*White blood cell count per bone and spleen. Mean ± SD are shown (*n* = 5)*.

### The Expression of CD122 and CD127 on CD8^+^ T Cells Is Similar between Different Bones

**Figure 2:**
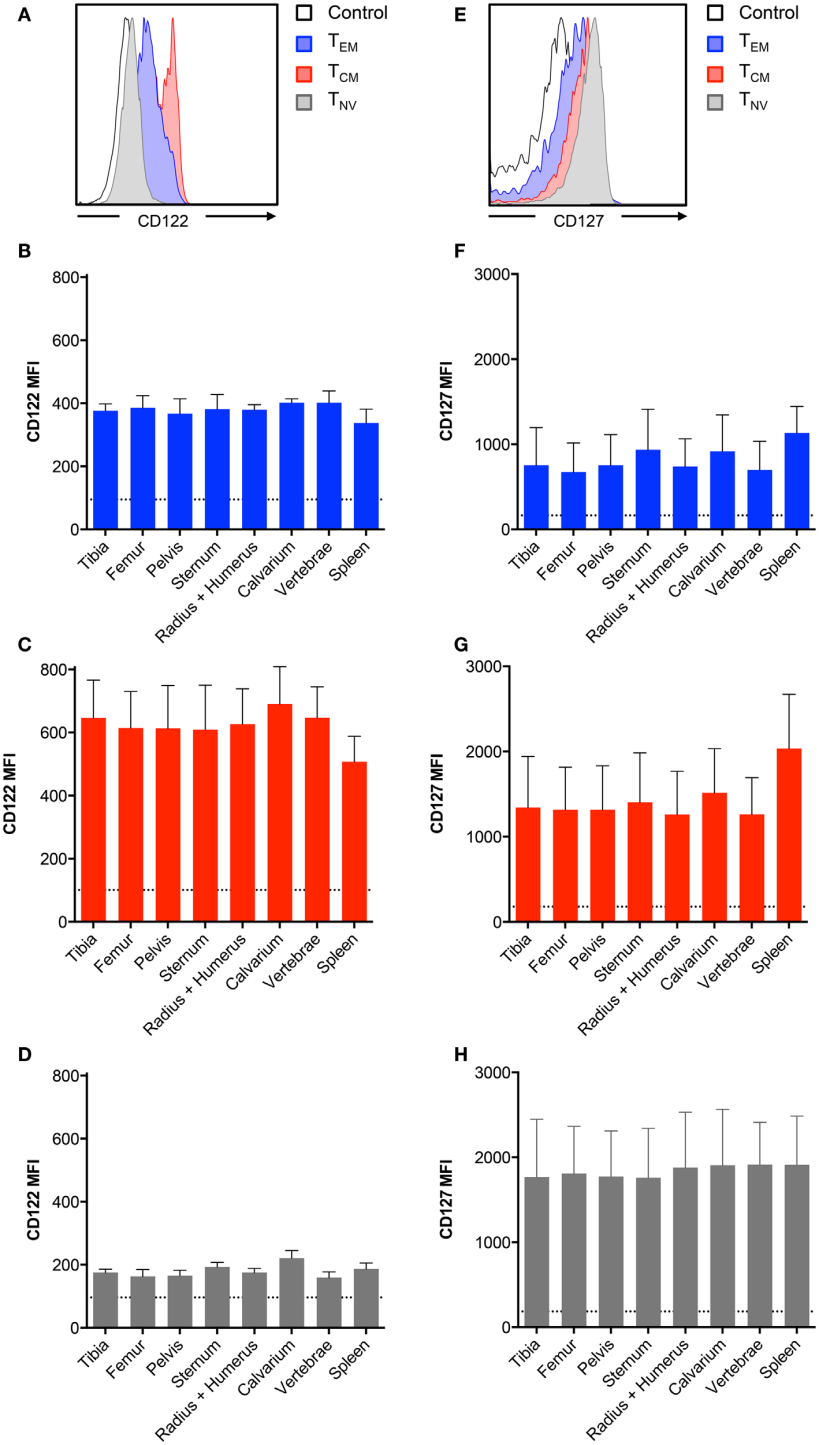
**BM memory CD8^+^ T cell subsets differ in their expression of survival receptors**. **(A,E)** Representative histograms showing expression of CD122 and CD127 in CD8^+^ T_EM_, T_CM_, and T_NV_ subsets. Black line indicates intensity of unstained cells. **(B–D)** MFI of CD122 or **(F–H)** MFI of CD127 on T_EM_, T_CM_, and T_NV_ subsets. Dotted line indicates MFI of unstained cells. Graphs show mean ± SD for each bone (*n* = 5–8).

The cytokines IL-7 and IL-15 are important for the development and maintenance of BM CD8^+^ T cells (11–13). In the murine BM, the expression of these cytokines is primarily confined to stromal cells (22, 23). As bones differ in structure, and possibly also in composition of IL-7 and IL-15 producing stromal cells, we tested if BM CD8^+^ T cells regulate the expression of the IL-7 and IL-15 receptors, according to their current location. This is particularly interesting, as IL-15 can regulate the expression of the receptor for IL-7 (24). Therefore, we compared the presence of CD122 and CD127 on CD8^+^ T cells collected from different bones. The highest expression of CD122 was found on the T_CM_ cells, whereas T_NV_ cells had the lowest expression of CD122 (Figure 2A). In contrast, T_NV_ cells expressed the highest levels of CD127, while T_EM_ cells expressed the lowest levels of CD127 (Figure 2E). Despite these marked differences in expression levels between the CD8^+^ T cell subsets, we observed similar expression of CD122 (Figures 2B–D) and CD127 (Figures 2F–H) by CD8^+^ T cells harvested from different bones. It remains to be investigated whether the lack of anatomical differences in respect to receptor levels also reflects similar concentrations of IL-7 and IL-15 in BM in different bones.

### BM Effector Memory CD8^+^ T Cells Strongly Increase after Infection with LCMV

**Figure 3:**
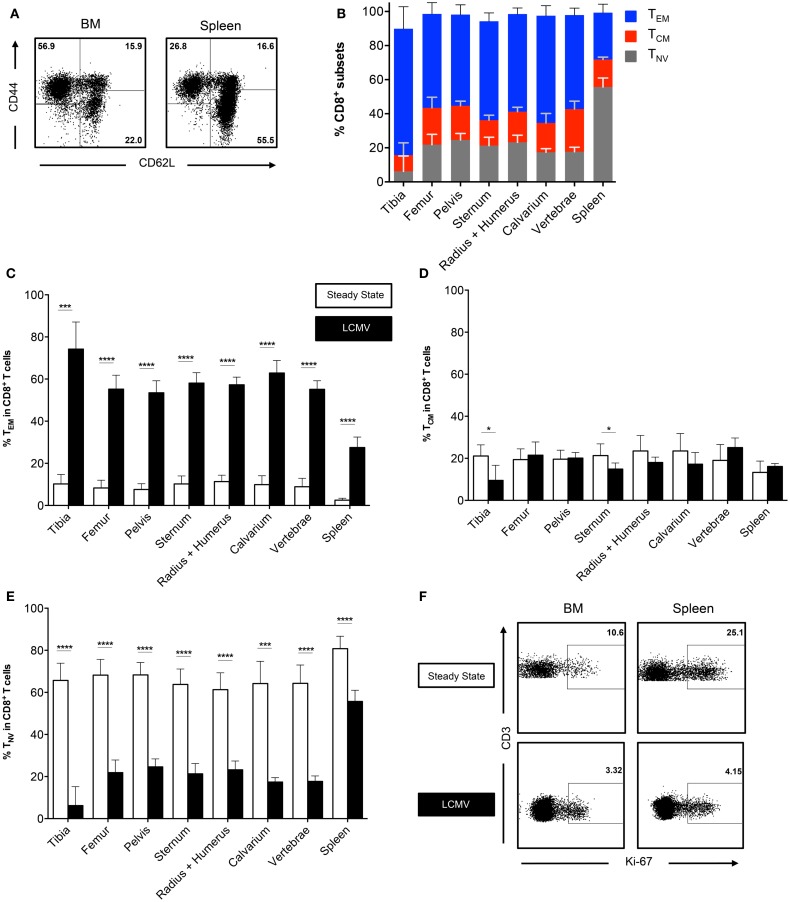
**The CD8^+^ T cell population consists primarily of memory cells after infection with LCMV**. **(A)** Representative FACS plots showing expression of CD44 and CD62L in CD8^+^ T cells for BM (tibia) and spleen. **(B)** Frequency of T_EM_, T_CM_, and T_NV_ subsets in CD8^+^ T cells. **(C–E)** Frequency of T_EM_, T_CM_, and T_NV_ subsets in the CD8^+^ T cell population during steady state and after infection with LCMV. White bars = steady state and black bars = LCMV. Data for **(C–E)** are identical to that for Figure 1D and **(B)**. **(F)** Representative FACS plots showing expression of Ki-67 on T_EM_ cells in spleen and BM during steady state (femur) and after infection with LCMV (vertebrae). Graphs show mean ± SD of each bone (*n* = 4–8), pooled from the three independent steady states and the two independent LCMV experiments. Statistical analysis was performed with unpaired *t* test followed by Welch’s correction. Significance is indicated by *p < 0.05, ***p < 0.001, and ****p < 0.0001.

We did not observe differences in CD8^+^ T cell frequencies in BM collected from different bones in the steady state. Thus, we questioned whether this would change after an infection that elicits a large influx of memory T cells. Therefore, we infected mice with the Armstrong strain of LCMV. This acute systemic infection is cleared from the BM within 8 days due to a strong CD8^+^ T cell response (25, 26). We analyzed the BM in the memory phase (>42 days) and found that the frequencies of CD3^+^ cells and CD8^+^ T cells were lower when compared to the spleen, but still similar between bones (data not shown). Interestingly, we found that the frequency of the CD8^+^ T_EM_ subset strongly increased, ranging from ~10% in the steady state to ~60% after LCMV (Figures 3A,B). The increase in T_EM_ cells corresponded with a decrease in T_NV_ cells, whereas the T_CM_ subset was largely unaffected (Figures 3C–E). This was comparable between all bones. The T_EM_ subset also increased in the spleen, although here the majority (~55%) of the CD8^+^ T cells still exhibited a naïve phenotype. Both BM and spleen T_EM_ cells were primarily Ki-67^−^, indicating that they are in the G0 phase of cell cycle, and are thus resting memory CD8^+^ T cells (Figure 3F). This was similar for all the different bones (data not shown). In summary, we show that even after resolved infection with LCMV, no anatomical differences occur in the BM regarding CD8^+^ T cell frequencies.

### BM Contains CD8^+^ T Cells with a Tissue-Resident Memory Phenotype

**Figure 4:**
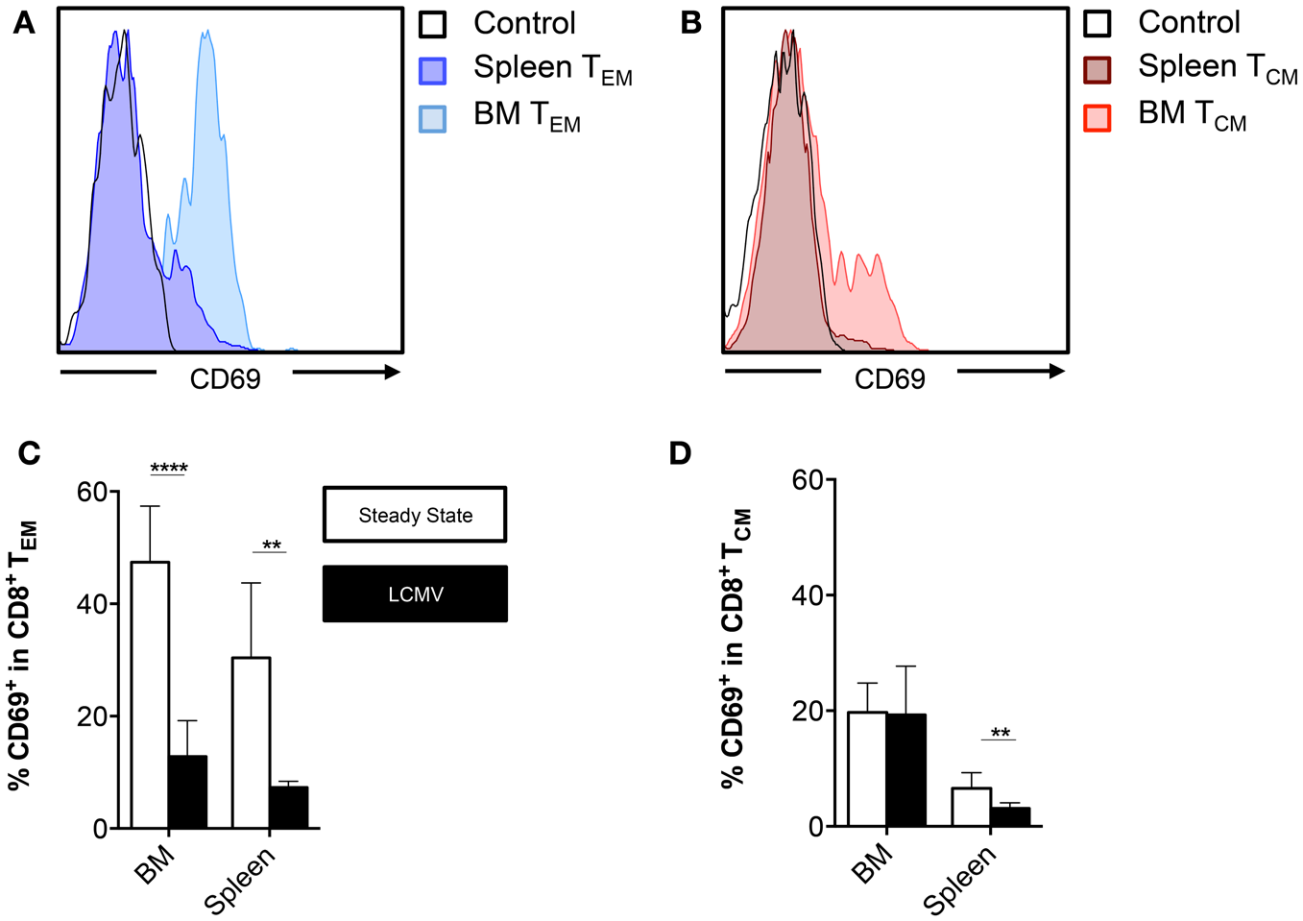
**BM memory CD8^+^ T cell subsets contain cells that express CD69**. **(A,B)** Representative histograms showing expression of CD69 on T_EM_ or T_CM_ cells in BM (femur) and spleen. **(C,D)** Frequency of CD69^+^ cells in the CD8^+^ T_EM_ or T_CM_ subsets in BM (vertebrae) and spleen. Graphs show mean ± SD of the vertebrae (*n* = 4–8), pooled from the three independent steady states and the two independent LCMV experiments. White bars = steady state and black bars = LCMV. Statistical analysis was performed with unpaired *t* test followed by Welch’s correction. Significance is indicated by **p < 0.01 and ****p < 0.0001.

Apart from being a primary and secondary lymphoid organ, BM, like any other tissue, is susceptible to viral infections (27, 28). Interestingly, over the past few years, it has been reported that following an infection, a subset of memory CD8^+^ T cells has the ability to take up residence in a particular tissue and provide tissue-specific immunity. These tissue-resident memory T cells (T_RM_) have been identified in skin, female genital tract, intestinal mucosa, kidney, pancreas, stomach, heart, salivary glands, and the brain (29–34). These cells are characterized by the expression of the C-type lectin CD69, which inhibits the expression of the egress receptor sphingosine-1-phosphate receptor 1 (S1PR1) (35, 36). Moreover, T_RM_ cells are identified by the absence of CD62L, making them a subgroup of the T_EM_ subset (37). Here, we examined the expression of CD69 by BM T_EM_ cells and compared these frequencies with the CD69 expression in other BM CD8^+^ T cell subsets. We found that during the steady state, approximately half (~47%) of BM T_EM_ cells and ~20% of BM T_CM_ expressed CD69 (Figures 4A,B). The T_NV_ subset barely expressed CD69 (data not shown). The frequencies of CD69 in each CD8^+^ T cell subset were similar for all bones (data not shown). Furthermore, the frequencies in the BM memory CD8^+^ T cells subsets were much higher than in the equivalent memory CD8^+^ T cell subsets located in the spleen (Figures 4A–D). Of specific note, after the infection with LCMV, the frequency of BM and spleen CD69^+^ cells decreased in the T_EM_ subset, while the frequency of BM CD69^+^ cells in the T_CM_ subset remained the same. However, we did not observe differences in absolute numbers of CD69^+^ T_EM_ cells before and after infection (data not shown). This indicates that the decreased frequency of CD69^+^ T_EM_ cells after infection results from a massive influx of CD69^−^ T_EM_ cells. In conclusion, BM contains a significant number of CD8^+^ T cells with a T_RM_ phenotype, though an LCMV infection elicits an influx of mostly conventional T_EM_ cells.

### BM Harbors a Significant Portion of LCMV-Specific CD8^+^ T Cells

**Figure 5:**
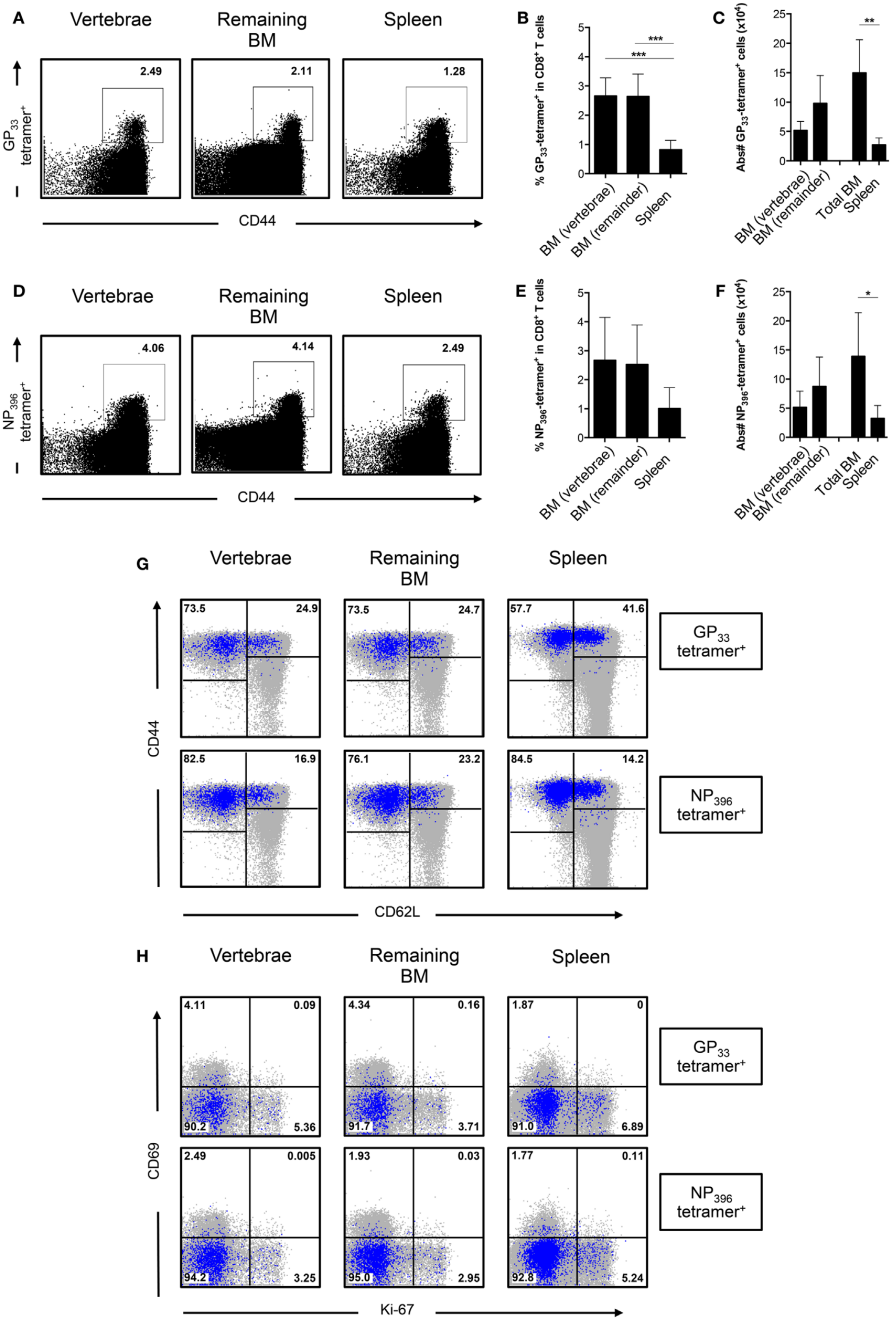
**The frequency of LCMV-specific CD8^+^ T cells is similar between BM in vertebrae and the remaining bones**. **(A)** Representative FACS plots showing staining of GP_33_-tetramer in CD8^+^ T cells. **(B)** Frequency and **(C)** absolute numbers of GP_33_-tetramer^+^ cells in CD8^+^ T cells. **(D)** Representative FACS plots showing staining of NP_396_-tetramer in CD8^+^ T cells. **(E)** Frequency and **(F)** absolute numbers of NP_396_-tetramer^+^ cells in CD8^+^ T cells. **(G)** Representative FACS plots showing expression of CD44 and CD62L of GP_33_-tetramer^+^ or NP_396_-tetramer^+^ CD8^+^ T cells. **(H)** Representative FACS plots showing expression of CD69 and Ki-67 of GP_33_-tetramer^+^ or NP_396_-tetramer^+^ CD8^+^ T cells. For **(G,H)**, tetramer^+^ cells (blue) are superimposed on all CD8^+^ T cells (gray). Percentages shown in **(G,H)** reflect the frequencies of tetramer^+^ CD8^+^ T cells. Graphs show mean ± SD of vertebrae or remaining BM (*n* = 3–6), pooled from the two independent experiments. Statistical analysis was performed with one-way ANOVA followed by Tukey’s correction or with unpaired *t* test followed by Welch’s correction. Significance is indicated by *p < 0.05, **p < 0.01, and ***p < 0.001.

In order to analyze CD8^+^ T cells generated specifically against LCMV, we pooled BM from different bones to obtain sufficient cell numbers (26). BM from the vertebrae was analyzed separately because of its high cellularity. We enriched for CD8^+^ T cells and stained with MHC-I tetramers loaded with the LCMV epitopes GP_33–41_ and NP_396–404_. We observed that the spleen had lower frequencies of LCMV-specific CD8^+^ T cells compared to either BM compartment (Figures 5A,D). We did not observe differences in frequencies of LCMV-specific CD8^+^ T cells between the BM from vertebrae and the other bones (Figures 5B,E). Based on the absolute numbers, the vertebrae itself contained ~35% of all GP_33_-tetramer^+^ and 37% of all NP_396_-tetramer^+^ CD8^+^ T cells present in the total BM. Furthermore, as we harvested the majority of the BM from the body, we could calculate that BM harbored 1.5 × 10^5^ GP_33_-tetramer^+^ and 1.4 × 10^5^ NP_396_-tetramer^+^ CD8^+^ T cells, which was fivefold to sixfold more than the spleen (Figures 5C,F), thereby emphasizing the role of the BM as memory T cell organ. LCMV-specific CD8^+^ T cells in BM primarily (~75%) had a T_EM_ phenotype, while the remainder exhibited a T_CM_ phenotype (Figure 5G). Additionally, LCMV-specific CD8^+^ T cells barely expressed CD69 or Ki-67, indicating that they were not actively cycling, but also not T_RM_ cells (Figure 5H). Our results suggest that the distribution of LCMV-specific CD8^+^ T cells is comparable between BM in the vertebrae and the rest of the bones, and that these cells are phenotypically similar to the rest of the memory CD8^+^ T cells. Our data also suggest that infection with LCMV does not result in substantial generation of LCMV-specific CD8^+^ T cells with a T_RM_ phenotype.

## Discussion

In the present study, we examined BM from murine tibia, femur, pelvis, sternum, radius, humerus, calvarium, and vertebrae and addressed if anatomical differences exist at the level of BM CD8^+^ T cells. Here, we show that during the steady state, BM derived from different bones had similar CD8^+^ T cell frequencies. Furthermore, the frequencies of the T_EM_, T_CM_, and T_NV_ subsets were also comparable between all the bones. We also examined BM during the memory phase of a LCMV infection. This virus is cleared primarily by CD8^+^ T cells and results in the generation of virus-specific memory CD8^+^ T cells, which remain detectable long after the initial infection (25, 26). Similarly to the steady state, we did not observe anatomical differences in BM after infection with LCMV. To date, only a limited number of studies have addressed the possible anatomical differences in the BM. These studies primarily focused on the functional differences within different regions inside a bone, but not necessarily between different bones. The majority of the studies found functional, but limited differences in frequencies of HSCs (3–5). It remains to be determined if BM T cells derived from different bones are also functionally distinct. Results obtained from a study performed with human BM suggest that this might not be the case. Pritz et al. (38) compared the phenotype and function of T cells derived from iliac crest and the femoral shaft and found no differences between the distribution of T cell populations and their cytokine production. Interestingly, although we found no differences between bones, we did observe that both during the steady state and after infection with LCMV, the majority of CD8^+^ T cells were located in the vertebrae, a collection of bones that has not been well studied and is not frequently included during sample preparation. From both a practical and ethical point of view, inclusion of the vertebrae can limit the amount of mice required for any given experiment, as it holds more than a third of all BM present in the murine body.

Here, we also demonstrated that BM substantially changes after infection with LCMV. The decline in frequency of T_NV_ cells coincided with the increased frequency of T_EM_ cells. As we did not observe differences in absolute numbers of total CD8^+^ T cells between steady state and LCMV-infected mice, our results suggest that the space in the BM is limited, resulting in one subset being replaced by another. Sercan Alp et al. (16) demonstrated that memory CD8^+^ T cells colocalize with IL-7-producing reticular stromal cells, in a 1:1 ratio in the BM. This, combined with our results and the fact that CD8^+^ T_NV_ cells primarily depend on IL-7 for survival [reviewed in Ref. (39)], suggests that CD8^+^ T_NV_ cells were outcompeted or blocked from entering these IL-7-rich niches, as these became occupied by memory CD8^+^ T cells. Yet, we show that BM memory CD8^+^ T cells express the receptors to respond to both IL-7 and IL-15, which could indicate that naïve and memory CD8^+^ T cells reside in different niches. If this is indeed the case, our results suggest that after infection with LCMV, the BM microenvironment changed and became less favorable for naïve CD8^+^ T cells and/or a more advantageous for memory CD8^+^ T cells. As LCMV has been shown to infect BM stromal fibroblast and endothelial cells (27), it could well be that the cellular sources of IL-7 and IL-15 in the BM are severely affected by the infection. Alternatively, there could also be a role for hematopoietic cells, as dendritic cells can increase their IL-15 production in response to inflammatory signals (40). Further studies are required to show how the BM niches that maintain CD8^+^ T cells adapt in order to accommodate the substantial amount of memory CD8^+^ T cells generated after infection with LCMV and how this relates to the spleen. Moreover, it remains unclear why the BM harbors so many T_EM_ cells long after the infection has been resolved and whether their presence affects the hematopoietic function of the BM, as activated immune cells have been shown to directly influence HSC function and hematopoiesis [reviewed in Ref. (41)].

Furthermore, we demonstrated that BM contained CD8^+^ T cells with a T_RM_ (CD44^+^CD62L^−^CD69^+^) phenotype. In the past, the surface molecule CD69 was associated with the “recently activated” status of T cells. More recently, this surface molecule has become important for its role in tissue retention, as it downregulates S1PR1 and thereby blocks the egress of lymphocytes from tissues (35). Currently, it is unclear if CD69 alone is sufficient for identification of T_RM_ cells in BM, as it has not been unequivocally demonstrated that CD69^+^ BM memory CD8^+^ T cells are non-circulating cells. Additionally, it has been postulated that T_RM_ cells may reside in the CD69^−^ fraction of memory T cells (42). Nonetheless, BM memory CD8^+^ T cells, which express CD69, resemble T_RM_ cells in other tissues, as they have low expression of S1PR1 (16). Furthermore, in accordance with our findings, BM memory CD8^+^ T cells are not activated, but rather quiescent in terms of proliferation and gene expression (16). It is thus highly likely that the CD69^+^ memory CD8^+^ T cells that we identified in BM are resident, rather than recently activated T cells. Currently, it is understood that T_RM_ cells reside in tissues where the initial infection took place and are positioned as the first line of defense, in order to accelerate pathogen elimination during secondary encounters (37). Whether T_RM_ cells in the BM are also strategically positioned to fulfill a similar function and protect the BM from invading pathogens remains to be investigated.

In summary, our findings suggest that in respect to the frequency of CD8^+^ T cells, BM harvested from one bone is representative of the BM found in all bones located throughout the body. Our results reinforce the notion that BM is a major immunological organ, as it is quantitatively superior to the spleen in accumulation and accommodation of memory CD8^+^ T cells, and should therefore be included when studying (adaptive) immune responses and memory T cell maintenance.

## Author Contributions

SG, SH, GB, and MP conducted the experiments; SG analyzed data; MP and MN supervised the project; and SG and MN wrote the manuscript.

## Conflict of Interest Statement

The authors declare that the research was conducted in the absence of any commercial or financial relationships that could be construed as a potential conflict of interest.
